# Postpartum Gray Matter Changes in the Auditory Cortex

**DOI:** 10.3390/jcm10235616

**Published:** 2021-11-29

**Authors:** Eileen Luders, Christian Gaser, Malin Gingnell, Jonas Engman, Inger Sundström Poromaa, Florian Kurth

**Affiliations:** 1 School of Psychology, University of Auckland, Auckland 1010, New Zealand; f.kurth@auckland.ac.nz; 2 Department of Women’s and Children’s Health, Uppsala University, 751 05 Uppsala, Sweden; malin.gingnell@akademiska.se (M.G.); inger.sundstrom@kbh.uu.se (I.S.P.); 3 Laboratory of Neuro Imaging, School of Medicine, University of Southern California, Los Angeles, CA 90089, USA; 4 Departments of Psychiatry and Neurology, Jena University Hospital, 07747 Jena, Germany; christian.gaser@uni-jena.de; 5 Department of Psychology, Uppsala University, 751 05 Uppsala, Sweden; jonas.engman@akademiska.se

**Keywords:** auditory cortex, brain plasticity, gray matter, MRI, postpartum, pregnancy

## Abstract

After giving birth, a mother’s brain undergoes functional adaptations fostering the ability to properly respond to the needs of her newborn. Tuning into and understanding her baby’s crying is among the top skills required and executed in the early stages of motherhood. However, surprisingly little is known about potential changes in the anatomy of the maternal auditory cortex. Therefore, in this longitudinal study, we compared the brains of 14 healthy women between immediate postpartum (within 1–2 days of childbirth) and late postpartum (at 4–6 weeks after childbirth), focusing on areas of the primary, secondary, and higher auditory cortex. We observed significant volume increases within all auditory regions and subregions examined, which might reflect rapid adaptations of the mother’s brain in relation to reliably interpreting her newborn’s cries. There was also a trend for a larger postpartum increase within right-hemispheric regions compared to left-hemispheric regions that might be specifically linked to the ability to discern the pitch, sound, and volume of a baby’s crying. Follow-up research is warranted to replicate these findings and evaluate their current interpretation.

## 1. Introduction

Mothers’ brains show links to their baby’s cries on a functional level. Scientific evidence for this natural phenomenon has been provided, for example, in neurophysiological experiments measuring brain activity in response to infant crying using functional magnetic resonance imaging or magnetoencephalography [1,2,3,4]. These studies have not only demonstrated that there is indeed an early crying-specialized activity in a mother’s brain, but also that the auditory cortex is among those regions that showed such an activity. Animal studies have provided insights into possible underlying mechanisms by pointing to the effect of hormones in the framework of infant-mother attachment [5]. Oxytocin in the auditory cortex of female mice, for example, increased the salience of acoustic social stimuli and, as such, enabled and enhanced pup retrieval behavior [6].

The question arises of whether the aforementioned functional, hormonal, and behavioral phenomena are accompanied by actual macroscopic changes within the auditory cortex. While at least two prior postpartum studies revealed gray matter increases throughout the brain that also encompassed the auditory cortex [7,8], structural imaging studies specifically targeting the maternal auditory cortex after giving birth are missing. Therefore, here we applied an advanced region-of-interest technique to gain further insights into possible physical changes of auditory brain regions when transitioning to motherhood. More specifically, we examined postpartum changes in brain tissue for three auditory main regions and five subregions, specifically Te1 consisting of Te1.0, Te1.1, and Te1.2 [9]; Te2 consisting of Te2.1 and Te2.2 [10]; and Te3 [11]. For the exact location and definition of these areas, please refer to [9,10,11]. Te1 constitutes the primary auditory cortex (or Brodmann Area 41); Te2 may be considered the secondary auditory cortex (or Brodmann Area 42); and Te3 constitutes part of the higher auditory cortex (corresponding to the posterior 2/3 of Brodmann Area 22). 

Aside from establishing which auditory (sub)regions will show changes—presumably increases rather than decreases, given the prior findings [7,8]—our study will reveal if there are different effects pertaining to left and right auditory regions. Such laterality effects are not unlikely. For example, prior analyses in mice have demonstrated that pup retrieval depends on the functional activity of the left ear, but not the right ear [6]. The same study also reported that retrieval behavior was accelerated by oxytocin and that oxytocin receptors were preferentially expressed in the left auditory cortex. Thus, any structural changes within auditory regions in the current study might be more pronounced in the left hemisphere than the right hemisphere. On the other hand, left Brodmann Area 22—as part of Wernicke’s area—has been implicated in language comprehension and speech production [12], whereas right Brodmann Area 22 is involved in discriminating pitch, sound, and volume. Thus, it seems equally possible that changes occurring during the first few weeks after giving birth are more pronounced in the right hemisphere than the left hemisphere, at least with respect to Te3.

## 2. Materials and Methods

Our sample included 14 healthy postpartum women, aged between 25 and 38 years (mean ± SD: 32.8 ± 4.0 years). All women were Caucasian, and 13 women (92.9%) were of Nordic origin. 13 women (92.9%) were married or cohabiting, and 11 women (78.6%) had an education on the university level. All women were recruited from the maternity ward at Uppsala University Hospital (Sweden) and had an uncomplicated delivery (*n* = 9 vaginal/*n* = 5 Caesarean); for 7 women (50%) it was a first-time delivery. For study exclusion criteria, please see [13]. All women underwent brain scanning at two time points, 1–2 days after delivery (immediate postpartum) and 4–6 weeks after delivery (late postpartum). All women provided written informed consent and all procedures were approved by the Regional Ethical Review Board, Uppsala (Sweden). Readers may seek access to the data through the corresponding author.

Brain images were acquired on a Philips Achieva 3T-X scanner using a phase-sensitive inversion recovery T1-weighted sequence and the following parameters: 5700 ms repetition time, 15 ms echo time, 400 ms inversion time, 90 degrees flip angle, 23 cm field of view, and 0.45 × 0.45 × 2.0 mm^3^ voxel size. Data processing was done in Matlab (http://www.mathworks.com/products/matlab, accessed on 22 October 2021) using the VBM8 toolbox (http://dbm.neuro.uni-jena.de/vbm, accessed on 22 October 2021) and a workflow optimized for longitudinal processing, as detailed elsewhere [13]. The procedure resulted in one image for each woman reflecting the difference in voxel-wise gray matter between immediate and late postpartum, with positive values indicating an increase and negative values a decrease. These difference images were multiplied with the cytoarchitectonic tissue probabilities for the regions of interest in each hemisphere (Te1.0, Te1.1, Te1.2, Te2.1, Te2.2, and Te3), as detailed elsewhere [14]. The required cytoarchitectonic probability maps are based on post mortem data [9,10,11,15] and freely available via EBRAINS (https://ebrains.eu/, accessed on 22 October 2021).

The region-specific volumetric changes were calculated in cubic millimeters (mm^3^) by adding the voxel-wise probability-weighted gray matter change across all voxels pertaining to each region of interest (Te1.0, Te1.1, Te1.2, Te2.1, Te2.2, and Te3). In addition, the total volume for the primary auditory cortex (Te1) was calculated as the sum of Te1.0, Te1.1, and Te1.2; the total volume for the secondary auditory cortex (Te2) was calculated as the sum of Te2.1 and Te2.2. One-sample t-tests were applied to detect possible volume changes after confirming that assumptions for parametric testing were met. Significance levels were adjusted for multiple comparisons by controlling the false discovery rate (FDR) [16,17]. Moreover, we calculated the regional changes in percent (%) and tested whether the changes in one hemisphere were larger than in the other. For this purpose, we applied paired t-tests using FDR-corrections, again after ensuring that assumptions for parametric testing were met. 

## 3. Results

**Figure 1:**
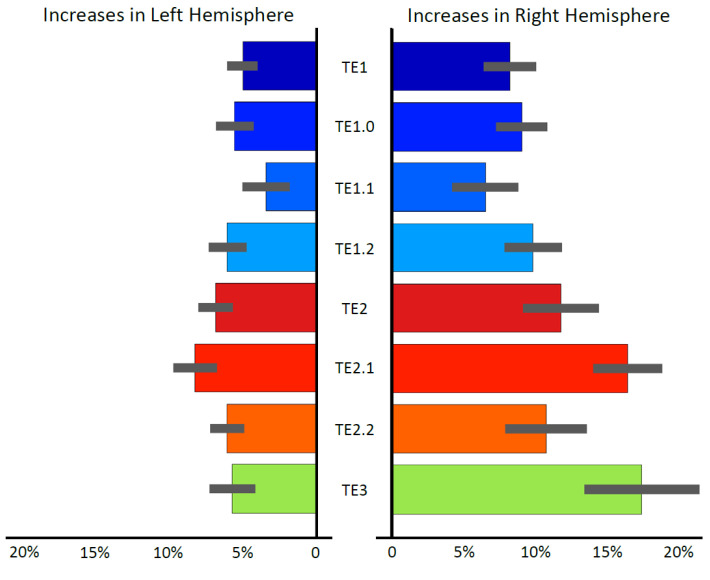
Changes in region-specific volumes between immediate and late postpartum. The colored bars show the mean increase in percent (%) in blue hues for Te1 (consisting of Te1.0, Te1.1, and Te1.2), in red hues for Te2 (consisting of Te2.1 and Te2.2), and in green for Te3. The smaller gray bars indicate the standard error of the mean.

There were no significant decreases between immediate postpartum (within 1–2 days of childbirth) and late postpartum (at 4–6 weeks after childbirth) for Te1, Te2, T3, or any of the subregions. In contrast, there were significant increases between these two time points for all regions of interest. Cohen’s d as well as statistical T- and FDR-corrected p-values (q values) are provided in Table 1; the region-specific changes (in %) are visualized in Figure 1. With respect to possible hemispheric differences, there was a trend for a significantly larger increase of right TE2.1 and right TE3 compared to left TE2.1 and left TE3 (both q ≤ 0.052).

## 4. Discussion

Contrasting the gray matter of the primary, secondary, and higher auditory cortex between immediate and late postpartum, we observed significant volume increases within all regions and subregions examined. In addition, there was a trend for a laterality effect within some regions, with larger postpartum increases within the right hemisphere compared to the left.

### 4.1. Direction and Location of the Effect

The direction of the effect (volume increases rather than decreases) after giving birth is in close agreement with the outcomes of most postpartum studies [7,8,13,18,19,20,21]; some of those studies [7,13,19,21] were conducted using the same dataset as in the current study. With respect to the location of the effect, our findings corroborate prior reports of gray matter increases in auditory areas, as detected when applying voxel-based morphometry (VBM), both in independent and overlapping samples [7,8]. Nevertheless, our current study also extends those findings based on VBM, which is most sensitive to effects in the size and shape of the selected smoothing kernel (spatial smoothing is a common processing step in VBM analyses). In other words, VBM studies are less sensitive with respect to effects that are non-spherically shaped or smaller/larger than the smoothing kernel (which was 8 mm or 12 mm FWHM in the aforementioned studies). Thus, by specifically focusing on—and distinguishing between—regions and subregions of the auditory cortex, our study provides substantial and additional evidence for postpartum tissue changes within primary, secondary, and higher auditory areas. As such, it may also inform future studies investigating potential changes in auditory processing and sound perception during pregnancy and after giving birth [22].

### 4.2. Possible Functional Links

Overall, our findings might reflect adaptations of the mother’s brain associated with listening for and interpreting her newborn’s cries as well as other idiosyncratic sounds (gurgles, grunts, whines, squeals, etc.). The detected changes on the macro-anatomical level (i.e., gray matter increases) may reflect events on the micro-anatomical level (i.e., angiogenesis, dendritogenesis, gliogenesis, or synaptogenesis) which may drive and/or result from syncing a mother’s brain to her newborn’s means of communicating. The functional significance of the observed auditory changes in the maternal brain might thus be to foster a mother’s ability to extract crucial information—not only in regards to physical and emotional states of her baby but also to degrees of urgency—in order to effectively intervene, altogether contributing to her newborn’s wellbeing and flourishing. In addition to understanding what the different sounds of her baby signify (e.g., being hungry, sleepy, too hot, too cold, wet, in pain etc.), postpartum changes within the auditory cortex may also allow a new mother to recognize her own baby’s cries [23], an ability reported to increase rapidly within a few days postpartum [24].

As discussed elsewhere, the maternal brain during the very early postpartum period constitutes an approximation of the pregnant brain [7,19]. Thus, apparent gray matter gain in auditory regions at late postpartum compared to immediate postpartum might also just reflect a restoration of brain tissue that may have been lost during pregnancy. Albeit research on this aspect is extremely sparse, one study [25] reported a significant gray matter decrease during pregnancy in various brain regions, including the left and right superior temporal gyrus, superior temporal sulcus, and middle temporal gyrus (i.e., regions that contain or are in close proximity to the auditory regions investigated in our study). In the same vein, our present findings within auditory regions might corroborate prior observations with respect to impaired or aberrant auditory functioning during pregnancy, that often resolve after giving birth, as reviewed in [22]. We have no record of auditory functioning whatsoever for our cohort, and we did not obtain a brain scan before pregnancy. However, it is certainly possible that tissue decreases within auditory regions took place during pregnancy—with or without noticeable impairment in auditory function—and simply reversed after giving birth and as such resulted in the detected tissue increases. 

Finally, of course, it is also conceivable that these two possible mechanisms, brain reorganization and tissue restoration, are not mutually exclusive: The observed gray matter gain within the primary, secondary, and higher auditory cortex may be due to reorganization in some regions and due to restoration in others; and yet another set of regions might be affected by both mechanisms, either simultaneously or sequentially.

### 4.3. Hemispheric Differences

Our study revealed a trend for larger postpartum increases within some right-hemispheric regions compared to left-hemispheric regions. These structural laterality effects seem to be in agreement with functional laterality effects where infant crying (and also laughing) elicited stronger activations in auditory areas of the right hemisphere than the left hemisphere [3]. The auditory area where the left-right difference was most pronounced in our study was TE3, which corresponds to the posterior part of Brodmann Area 22. Since Brodmann Area 22 in the right hemisphere is closely involved in pitch, sound, and volume discrimination—while generating and understanding words are attributed to the left hemisphere (i.e., Wernicke’s area)—the observed laterality trend might be related to the mother’s ability to discern pitch, sound, and volume of her baby’s crying. Future studies aiming to replicate this asymmetric growth effect in the very early postpartum stages may additionally consider investigating if the rightward growth becomes less pronounced (or even negated by a leftward growth) as the child transitions from crying to cooing to babbling to talking.

## 5. Conclusions

Our study provides evidence for postpartum tissue increases within primary, secondary, and higher auditory areas. The observed effects may reflect adaptations of the mother’s brain in relation to interpreting her newborn’s cries. However, given the small sample size and the relatively sparseness of studies in this field of research, follow-up research is clearly warranted to replicate (and potentially extent) the present findings as well as to evaluate their current interpretation.

## Figures and Tables

**Table 1:** Changes in region-specific volumes between immediate and late postpartum.

Region	Left Hemisphere			Right Hemisphere		
	Cohen’s d	T	q ^1^	Cohen’s d	T	q ^1^
Te1	2.9346	5.2904	0.0002 *	2.5234	4.5491	0.0004 *
Te1.1	2.5349	4.5699	0.0004 *	2.9113	5.2485	0.0002 *
Te1.0	1.214	2.1885	0.0237 *	1.4394	2.595	0.0118 *
Te1.2	2.6764	4.8249	0.0003 *	2.7033	4.8734	0.0003 *
Te2	3.6535	6.5864	0.0001 *	2.5808	4.6527	0.0004 *
Te2.1	3.1287	5.6404	0.0002 *	3.9615	7.1416	0.0001 *
TE2.2	3.5196	6.3451	0.0001 *	2.1455	3.8679	0.0012 *
Te3	2.078	3.7462	0.0014 *	2.7147	4.894	0.0003 *

^1^ q-values are FDR-corrected *p*-values; * denotes significance.

## Data Availability

The conditions of our ethics approval do not permit public archiving of anonymized study data. Readers seeking access to the data should contact the corresponding author. Access may be granted to named individuals after completion of a formal data sharing agreement in accordance with ethical procedures governing the reuse of sensitive data.
